# Immunomodulators and Their Applications in Dentistry and Periodontics: A Comprehensive Review

**DOI:** 10.7759/cureus.46653

**Published:** 2023-10-07

**Authors:** Abhishek Pahade, Pavan Bajaj, Amit Reche, Unnati Shirbhate

**Affiliations:** 1 Periodontics, Sharad Pawar Dental College, Datta Meghe Institute of Higher Education and Research, Wardha, IND; 2 Public Health Dentistry, Sharad Pawar Dental College, Datta Meghe Institute of Higher Education and Research, Wardha, IND

**Keywords:** inflammation, oral health, immunomodulators, periodontics, dentistry

## Abstract

The intricate interplay between the immune system and oral health has revealed opportunities for novel therapeutic interventions in dentistry and periodontics. This review article delves into the pivotal role of immunomodulators in orchestrating immune responses within the oral cavity and their applications in managing various oral and periodontal conditions. The oral environment faces many challenges, from microbial infections to tissue injuries, necessitating a precise immune response for optimal oral health maintenance. Characterized by their ability to modulate immune reactions, immunomodulators emerge as versatile tools for maintaining immune equilibrium. This is a comprehensive overview of the mechanisms through which immunomodulators exert their effects, shedding light on their dual role as regulators of both pro-inflammatory and anti-inflammatory pathways. The diverse applications of immunomodulators within dentistry are explored in depth. Immunomodulators exhibit promising outcomes from managing common oral conditions like gingivitis, periodontitis, and oral ulcers to enhancing the integration of dental implants and promoting wound healing post-surgery. This article highlights the various types of immunomodulatory agents utilized in dental practice, elucidating their mechanisms of action, routes of administration, dosages, and potential side effects.

## Introduction and background

The field of dentistry has evolved significantly over the years, encompassing not only traditional oral health practices but also incorporating advancements in medical science. One such area of exploration and innovation is using immunomodulators in periodontics and dentistry. Immunomodulators, substances that exert a regulatory influence on the immune system, have emerged as valuable tools in managing various oral health conditions characterized by inflammation, immune dysregulation, and tissue damage [1].

Periodontitis is a prevalent dental disease characterized by an inflammatory cascade that directly impacts the periodontal tissues. This inflammatory upheaval is manifested through the inflammation of periodontal soft tissues and the gradual erosion of periodontal ligaments and alveolar bone [1]. Periodontitis has established intricate links with various systemic and distal inflammatory disorders. This web of connections intertwines periodontitis with conditions such as diabetes, cardiovascular disease, rheumatoid arthritis, and metabolic syndrome [2-4].

Beyond periodontal diseases, immunomodulators find application in various other aspects of dentistry. Oral mucosal conditions, like lichen planus and recurrent aphthous stomatitis, involve immune-mediated processes that lead to discomfort and compromised oral health [5]. Immunomodulatory interventions have demonstrated potential in alleviating symptoms and restoring mucosal integrity by regulating immune responses. Implant dentistry has also witnessed the integration of immunomodulatory approaches [6]. In oral cancer, immunomodulation has emerged as a promising avenue for enhancing the body's immune surveillance against malignant cells. Immunotherapies hold the potential to augment the immune response against oral cancer, opening new avenues for comprehensive treatment strategies [5,6].

## Review

Search methodology

**Figure 1:**
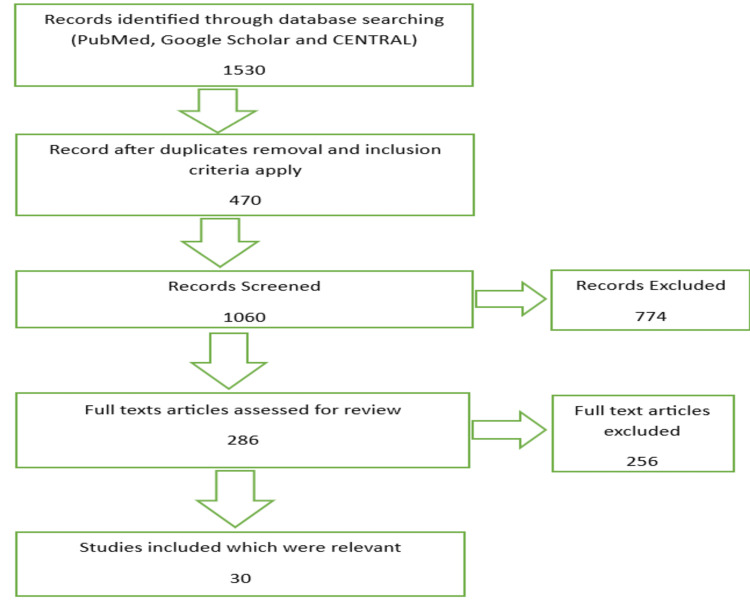
PRISMA flow diagram Adopted from the Preferred Reporting Items for Systematic Reviews and Meta-Analyses (PRISMA)

The articles were searched in databases such as PubMed, Google Scholar, and Scopus for the review work to primarily published studies from July 2022 using Medical Subject Headings (Mesh) terms and keywords "Dentistry, Periodontics, Immunomodulators, Oral health, Inflammation". The publications on the role of immunomodulators in periodontics were included in research work in research surveys, experimental surveys, and review publications. The relevant data is gathered and carefully examined related to immunomodulators in periodontics, applications, and their mechanism of action advantages. We curated data about our topic, excluded 256 irrelevant articles based on immunotherapy only, and included 30 articles that showed clinical aspects based on immunotherapy applications in dentistry and periodontology with relevant studies, reports, and reviews. Figure 1 shows the Preferred Reporting Items for Systematic Reviews and Meta-Analyses (PRISMA) diagram with search database information.

Immune response in oral health

The concept of "immunity" encapsulates the innate and acquired capacity of the host to withstand and defend against harm inflicted by microorganisms and the substances they produce [2,5]. The oral immune system encompasses a range of specialized cells, signaling molecules, and immune mediators collaborating to provide a dynamic defense against potential threats. Epithelial cells lining the oral mucosa act as the first line of defense by forming a physical barrier and producing antimicrobial peptides [7,8]. Beneath this layer, immune cells such as neutrophils, macrophages, dendritic cells, and thymus lymphocytes (T lymphocytes) are strategically positioned to detect and respond to invading pathogens [7-10]

Numerous immunological disorders exert their intricate pathogenic influence within the confines of the oral cavity. Within this realm, the application of steroids, the stalwart of treatment, stands to address these ailments. Yet, the extended use of steroids harbors its adversities, and scenarios arise where their efficacy falters against the complexities of involuted pathogenesis. The call emerges to include immunomodulatory agents [5].

"Immuno" finds its roots in the immune response, the intricate orchestration of our immune system, while "modulation" embodies finely calibrating according to optimal proportions. Thus, immunomodulators, whether borne from nature or crafted synthetically, assume the role of regulators, harmonizing the immune system's cadence. This recalibration extends to reducing inflammatory replication, allowing a more tempered immune response [5].

Immunomodulators

Immunomodulators encompass a spectrum of internal and external entities with the remarkable capacity to shape and adjust the immune response parameters. These agents wield power to influence immune reaction's breadth, nature, duration, and effectiveness, effectively sculpting the intricate dance of our body's defense mechanisms [8]. Immunomodulators aim to optimize immune responses by enhancing the immune defense against infections or dampening excessive immune reactions that could lead to tissue damage [9].

The immunological microenvironment is drastically altered by periodontitis. Leukocyte and inflammatory molecule activity rise, which can reduce inflammation, but this overactive state may significantly damage alveolar bone and other periodontal tissues. A promising approach for treating periodontitis is to modify the immunological microenvironment. To achieve higher levels of immune control and tissue repair, new anti-inflammatory and periodontal regeneration therapies can improve the immune environment and stimulate cell homing and tissue repair [7,9].

Immunosuppressants

Immunosuppressants are a subset of immunomodulators that act to dampen or suppress the immune response. They are used primarily to reduce immune activity in conditions where an overactive immune system leads to tissue damage, inflammation, or autoimmune reactions. Immunosuppressants are often employed in organ transplantation to prevent rejection, as well as in the treatment of autoimmune diseases and allergic reactions. By inhibiting immune cell activation, cytokine production, or immune signaling pathways, immunosuppressants help restore balance and prevent immune-mediated harm [10]. Immunosuppressants include cyclosporine A, tacrolimus, azathioprine, mofetil mycophenolate, prednisone, and muromonab [10].

Immunostimulants

Immunostimulants are immunomodulators that enhance or stimulate the immune response. They are used when the immune system's activity needs to be bolstered to combat infections or certain cancers. Immunostimulants can amplify immune cells' function, increase cytokine production, and improve antigen presentation. They are employed to support the body's natural defenses and enhance the efficacy of vaccines. In cancer treatment, immunostimulants can activate the immune system to recognize and attack cancer cells, leading to promising developments in immunotherapy [6,10]. Examples of immunostimulants are interferon-gamma, interleukin-2 (IL-2), Bacillus Calmette-Guérin (BCG), lipopolysaccharides (LPS), glucan, and isoprinosine [10].

Mechanisms

The mechanisms through which immunomodulators exert their influence are intricate and diverse. At the core of their functionality lies the ability to interact with immune cells, silicon molecules, and pathways to direct immune responses toward desired outcomes. These mechanisms can be broadly categorized into pro-inflammatory and anti-inflammatory modulations [8,11]. In addition to protecting against periodontopathic bacteria and their byproducts from entering gingival tissues, the gingival epithelial cells are crucial cellular elements of the innate immune system in the gingival sulcus. Antimicrobial compounds are vital in innate immunity therapeutic adjuvants in treating oral infections, including periodontal disease, and limiting the plaque microbiota within the gingival sulcus. The quick and severe progression of periodontal damage under settings of PMN malfunction provides evidence for the critical role of the PMN barrier function in maintaining periodontal health [7,8]. Mesenchymal stem cells (MSCs) have a potent immunomodulatory effect, making them desirable for tissue regeneration. MSCs obtained from dental tissue can be isolated from various tissues, including the gingiva, periodontal ligament, deciduous teeth, apical papilla, and dental pulp. Numerous in vitro studies have shown that the effect of dental MSCs on immune cells may vary depending on the experimental setting, the origin of the MSC tissue, and the kind of immune cell preparation [9,12]. Most research has demonstrated that immune cells that have been activated dramatically upregulate the immunomodulatory activity of dental MSCs. MSCs primarily have immunosuppressive properties, which reduce the activation of immune cells [12].

Pro-Inflammatory Modulation

Immunomodulators can bolster pro-inflammatory pathways in scenarios demanding a robust immune response, such as combating infections. By stimulating immune cells like macrophages and neutrophils, cytokines and chemokines are amplified, recruiting more immune defenders to the battleground. This augmentation heightens the antimicrobial activity and reinforces the immune system's armor [7].

Anti-Inflammatory Modulation

Alternatively, immunomodulators function as pacifiers when inflammation threatens to spiral out of control. They quell the fiery cascade of immune reactions by inhibiting the activity of pro-inflammatory cytokines like interleukins and tumor necrosis factor-alpha (TNF-α). This dampening effect curtails tissue damage and supports the resolution of inflammation, preventing collateral harm [7].

Cytokine Balancing in T Regulatory Cells

Immunomodulators can regulate regulatory T cells (Tregs), a specialized subset of T lymphocytes responsible for maintaining immune tolerance and preventing autoimmunity. By augmenting Treg populations, immunomodulators instill restraint within the immune response, preventing hyperactivity and inflammation [7].

Cytokine Receptor Modulation

Another mechanism involves modulating the interactions between cytokines and their receptors. Immunomodulators can enhance or inhibit these interactions, dictating the balance between pro-inflammatory and anti-inflammatory signaling. This manipulation yields control over the immune response's intensity and direction [7].

Periodontitis immunotherapy

Table 1 enumerates the periodontal immunotherapies' detailed classification, including drug therapy, microbial therapy, stem cell therapy, gene therapy, and other therapies [9-11].

**Table 1:** Periodontitis immunotherapy Created by Author PDLSCs, periodontal ligament stem cells; GMSCs, gingival mesenchymal stem cells; MSCs, mesenchymal stem cells; PT, periodontal therapy; PDT, photodynamic therapy; ICG, indocyanine green; PTT, photothermal therapy; MB-PDT, methylene blue-mediated photodynamic therapy; LIPUS, low-intensity pulsed ultrasound

Drug therapy	Microbial therapy	Stem cell therapy	Gene therapy	Other therapies
Targeting neutrophils	Probiotic therapy	PDLSCs		PT-associated with PDT
Targeting monocytes	Antibacterial therapy	GMSCs		ICG-diode laser-based PTT
Targeting macrophages		Other MSCs		MB-PDT
Targeting T lymphocytes				Single-photon treatment
Targeting cytokines				LIPUS

Clinical applications of immunomodulators

In some instances, the immune response within the oral cavity can become hyperactive, leading to inflammatory conditions such as periodontitis, where the immune system's response to bacterial infection results in tissue damage. Immunosuppressants are utilized to mitigate this excessive immune response and prevent further harm. Glucocorticoids (corticosteroids), tacrolimus and cyclosporine, azathioprine, and methotrexate are some specific applications of common immunosuppressants in this field [10]. Topical or systemic corticosteroids are used to manage painful oral ulcerations, such as aphthous ulcers or oral lichen planus, by reducing inflammation and promoting healing [11-13]. Corticosteroids can be prescribed for managing acute or chronic oral inflammatory conditions, including allergic reactions and autoimmune disorders affecting the oral mucosa [5,10,11]. Topical formulations of tacrolimus and cyclosporine treat oral lichen planus, an autoimmune disease affecting the oral mucosa. They help suppress the immune response and reduce inflammation. These drugs may be considered for managing mucous membrane pemphigoid, an autoimmune blistering disorder affecting the oral mucosa [5,11]. These immunosuppressants can be employed in severe oral mucosal diseases, such as pemphigus vulgaris or pemphigoid, to control immune-mediated damage to oral tissues. Methotrexate may be used for patients with recurrent aphthous ulcers refractory to other treatments. It modulates immune responses to reduce ulcer frequency and severity [10,11,14].

On the other hand, there are scenarios where the immune response needs enhancement to combat infections and promote healing. In dentistry, immunostimulants can augment the immune system's defenses in oral surgery, dental implant procedures, and wound healing after extractions. Localized applications of immunostimulants can accelerate tissue repair and minimize the risk of post-operative infections. Moreover, in periodontics, immunostimulatory agents can bolster the immune response in managing gum infections and promoting tissue regeneration. Levamisole, thalidomide, diamino diphenyl sulphone (DAPSONE), efalizumab, and BCG are some specific applications of common immunostimulants in this field [15-18]. Levamisole, an immunostimulant, has been used to enhance the immune response and improve the healing of oral lichen planus, an autoimmune disorder affecting the oral mucosa [5,11,19]. Thalidomide has shown promise in treating recurrent aphthous ulcers. It modulates immune responses and can help reduce the frequency and severity of these oral ulcers [5,11]. Dapsone, an immunostimulant, is sometimes used to manage mucous membrane pemphigoid, an autoimmune blistering disorder affecting the oral mucosa [5,11]. Efalizumab is an immunostimulatory monoclonal antibody used to treat severe psoriasis. Its potential applications in oral lichen planus therapy involve modulating the immune response to manage inflammation and promote healing [10,11]. BCG, a vaccine used for tuberculosis, has been investigated as an immunostimulant in oral cancer treatment. Local administration of BCG can enhance the immune response to target and destroy cancer cells [11].

Immunotolerance in dental procedures

Materials like metals (e.g., titanium) are introduced to the oral environment in specific dental procedures, such as orthodontic treatments or dental implant placements [5,20,21]. These foreign materials have the potential to trigger immune responses, leading to complications such as inflammation or implant rejection. Immunotolerance strategies aim to minimize the immune response against these materials, allowing for better integration and long-term success. This can involve immunomodulators to prevent excessive immune reactions, promoting better compatibility between the foreign material and the host's immune system [5,22-25].

Microbial therapies in periodontics

Microbial therapies, also known as microbiome-based therapies or microbial modulation, are emerging as innovative approaches in periodontics. These therapies recognize the integral role of the oral microbiome, the diverse community of microorganisms residing in the oral cavity, in maintaining oral health and its potential role in the development of periodontal diseases [26].

Probiotic Therapy: Balancing the Microbial Ecosystem

Probiotic therapy has garnered significant attention due to its potential to restore and maintain microbial balance within the body. In dentistry, this approach has profound implications for oral health. By introducing beneficial microorganisms, probiotics can counteract the proliferation of pathogenic bacteria, contributing to the prevention and management of oral diseases [7,27,28]. A balanced diet, smoking, poor oral hygiene, and modern lifestyle choices significantly upset the oral microbiome's sensitive equilibrium and trigger the onset of oral diseases like periodontitis, particularly in people genetically and epigenetically predisposed to them. By harnessing naturally occurring, helpful bacteria that are often present in healthy mouths to provide a natural defense against those bacteria that are regarded to be harmful to teeth and gums, probiotic technology promises a new approach to maintaining oral health [28]. 

Oral Health Applications of Probiotics

In dentistry, probiotic therapy can be applied to oral conditions such as periodontal diseases and caries. When administered through mouthwashes, lozenges, or chewing gums, probiotic strains can competitively exclude harmful bacteria, influence microbial metabolism, and modulate local immune responses. This can reduce inflammation, improve tissue healing, and enhance protection against cariogenic bacteria [7,28].

Antibacterial Therapy: Targeting Pathogenic Microorganisms

Antibacterial therapy, a well-established approach in medicine, has found renewed relevance in microbial treatment. However, the emphasis has shifted toward precision targeting, minimizing collateral damage to beneficial microbes, and combating antibiotic resistance [7,27]. By eradicating subgingival microorganisms still present after traditional mechanical periodontal therapy, systemic periodontal antibiotic therapy seeks to supplement mechanical periodontal treatment and boost the host defense system in overcoming the infection. [29].

Strategic Antibacterial Approaches in Dentistry

In dentistry, antibacterial therapy plays a pivotal role in managing oral infections and preventing the progression of periodontal diseases. Antibacterial agents can control disease and promote tissue regeneration by explicitly targeting pathogenic bacteria responsible for inflammation and tissue destruction. Emerging techniques, such as targeted antimicrobial peptides, aim to selectively neutralize harmful bacteria without disturbing the balance of the oral microbiome [7,27,30].

Future perspectives

The application of these therapies will continue to be shaped by advancements in personalized medicine, diagnostic techniques, and innovative drug delivery systems. However, navigating these new frontiers responsibly is crucial, ensuring rigorous research, evidence-based practice, and patient-centered care. With interdisciplinary collaboration, ethical considerations, and a commitment to advancing oral health, the future holds the potential for a paradigm shift in how oral diseases are approached and treated, ultimately leading to improved patient outcomes and a reimagined standard of care [28-30].

## Conclusions

In conclusion, integrating immunomodulators and microbial therapies in periodontics and dentistry represents a transformative shift toward personalized and targeted interventions. These innovative approaches recognize the intricate interplay between the immune system, oral microbiome, and oral health. By modulating immune responses, immunomodulators offer a nuanced way to manage inflammatory conditions and promote tissue regeneration. Meanwhile, microbial therapies, such as probiotics and targeted antibiotics, promise to restore microbial balance and prevent oral diseases by leveraging the symbiotic relationships within the oral microbiome. The effects of various forms of immune regulation on the periodontal microenvironment and periodontal tissue regeneration, including immune response patterns and cytokine networks in periodontal tissue in both healthy and inflammatory conditions, need to be further studied, similar to immunotherapy for other diseases.

## References

[1] Hajishengallis G, Korostoff JM (2017). Revisiting the Page & Schroeder model: the good, the bad and the unknowns in the periodontal host response 40 years later. Periodontol 2000.

[2] Hajishengallis G (2015). Periodontitis: from microbial immune subversion to systemic inflammation. Nat Rev Immunol.

[3] Konig MF, Abusleme L, Reinholdt J (2016). Aggregatibacter actinomycetemcomitans-induced hypercitrullination links periodontal infection to autoimmunity in rheumatoid arthritis. Sci Transl Med.

[4] Lamster IB, Pagan M (2017). Periodontal disease and the metabolic syndrome. Int Dent J.

[5] Peeyush S, Lata S, Ankur S, Shruti P, Monu Y (2015). Role of immunomodulators in oral diseases. J Int Dent Medical Res.

[6] Kondoh N, Mizuno-Kamiya M, Umemura N (2019). Immunomodulatory aspects in the progression and treatment of oral malignancy. Jpn Dent Sci Rev.

[7] Yang B, Pang X, Li Z, Chen Z, Wang Y (2021). Immunomodulation in the treatment of periodontitis: progress and perspectives. Front Immunol.

[8] Lebish IJ, Moraski RM (1987). Mechanisms of immunomodulation by drugs. Toxicol Pathol.

[9] Su Q, Alan Z, Ryan L, Gregory S (2022). Immunomodulation—what to modulate and why? potential immune targets. Front Dent Med.

[10] Bascones-Martinez A, Mattila R, Gomez-Font R, Meurman JH (2014). Immunomodulatory drugs: oral and systemic adverse effects. Med Oral Patol Oral Cir Bucal.

[11] Nikhil P (2023). Role of immunomodulators in oral diseases. Health and Medicine.

[12] Oleh A, Christian B, Alice B, Xiaohui F (2019). Immunomodulatory properties of dental-derived mesenchymal stem cells. Periodontology and Dental Implantology.

[13] Zhang X, Hasani-Sadrabadi MM, Zarubova J (2022). Immunomodulatory microneedle patch for periodontal tissue regeneration. Matter.

[14] Lakshita S, Sindhu R, Bharathwaj V (2023). Therapeutic effects of “Septilin” (herbal immunomodulator) against periodontal manifestations: a systemic review. J Surv Fish Sci.

[15] Vasovic M, Gajovic N, Brajkovic D, Jovanovic M, Zdravkovaic N, Kanjevac T (2016). The relationship between the immune system and oral manifestations of inflammatory bowel disease: a review. Cent Eur J Immunol.

[16] Ptasiewicz M, Grywalska E, Mertowska P, Korona-Głowniak I, Poniewierska-Baran A, Niedźwiedzka-Rystwej P, Chałas R (2022). Armed to the teeth-the oral mucosa immunity system and microbiota. Int J Mol Sci.

[17] Angelica Balingit and Jill Seladi-Schulman (2022 (2023). What are immunomodulators? healthline angelica balingit and jill seladi-schulman. https://www.healthline.com/health/immunomodulators.

[18] Duttenhoefer F, Fuessinger MA, Beckmann Y, Schmelzeisen R, Groetz KA, Boeker M (2019). Dental implants in immunocompromised patients: a systematic review and meta-analysis. Int J Implant Dent.

[19] Milad B, Faraz R, Reyhaneh H, Mehdi Y, Hossein K (2020). Immunological aspects of dental rejection. Biomed Res Int.

[20] Dong J, Wang W, Zhou W (2022). Immunomodulatory biomaterials for implant-associated infections: from conventional to advanced therapeutic strategies. Biomater Res.

[21] John T Immunity in the oral cavity. BiteSized Immunology: Organs & Tissues.

[22] Dr. DeAngelis (2019 (2023). Dr deangelis common types of dental diseases. http://Dr.DeAngelis.

[23] Quach H, Ritchie D, Stewart AK, Neeson P, Harrison S, Smyth MJ, Prince HM (2010). Mechanism of action of immunomodulatory drugs (IMiDS) in multiple myeloma. Leukemia.

[24] Kim JH, Kim DH, Jo S, Cho MJ, Cho YR, Lee YJ, Byun S (2022). Immunomodulatory functional foods and their molecular mechanisms. Exp Mol Med.

[25] Sandeep P, Runuk S, Sonal V, Chaitanya N (2012). Rationale in usage of immunomodulators for management of head, face and neck cancers. Int J Head Neck Surg.

[26] Allaker RP, Douglas CW (2009). Novel anti-microbial therapies for dental plaque-related diseases. Int J Antimicrob Agents.

[27] Ahmadi H, Ebrahimi A, Ahmadi F (2021). Antibiotic therapy in dentistry. Int J Dent.

[28] Baker JL, Edlund A (2018). Exploiting the oral microbiome to prevent tooth decay: has evolution already provided the best tools?. Front Microbiol.

[29] Muhammad H, Shahabe A, Talib N, Mohammad B (2018). Microbial etiology and antimicrobial therapy of peri-implantitis: a comprehensive review. Open Dent J.

[30] Kang Y, Sun B, Chen Y, Lou Y, Zheng M, Li Z (2021). Dental plaque microbial resistomes of periodontal health and disease and their changes after scaling and root planning therapy. mSphere.

